# Safety and efficacy of peripheral nerve blocks to treat refractory headaches after aneurysmal subarachnoid hemorrhage – A pilot observational study

**DOI:** 10.3389/fneur.2023.1122384

**Published:** 2023-04-20

**Authors:** Swarna Rajagopalan, Nanda Siva, Andrew Novak, Jeffrey Garavaglia, Casey Jelsema

**Affiliations:** ^1^Department of Neurology, Cooper Medical School of Rowan University, Camden, NJ, United States; ^2^West Virginia University School of Medicine, Morgantown, WV, United States; ^3^Department of Neurology, West Virginia University School of Medicine, Morgantown, WV, United States; ^4^Department of Pharmacy, West Virginia University School of Medicine, Morgantown, WV, United States; ^5^Department of Statistics and Data Analytics, Sandia National Laboratories, Albuquerque, NM, United States

**Keywords:** Headache, subarachnoid hemorrhage, peripheral nerve block, pain, opioid-sparing analgesia

## Abstract

**Objectives:**

Headache after aneurysmal subarachnoid hemorrhage (HASH) is common, severe, and often refractory to conventional treatments. Current treatment standards include medications including opioids, until the pain is mitigated. Peripheral nerve blocks (PNBs) may be an effective therapeutic option for HASH. We conducted a small before-and-after study of PNBs to determine safety, feasibility, and efficacy in treatment of HASH.

**Methods:**

We conducted a pilot before-and-after observational study and collected data for 5 patients in a retrospective control group and 5 patients in a prospective intervention PNB group over a 12-month period. All patients received a standard treatment of medications including acetaminophen, magnesium, gabapentin, dexamethasone and anti-spasmodics or anti-emetics as needed. Patients in the intervention group received bilateral greater occipital, lesser occipital, and supraorbital PNBs in addition to medications. The primary outcome was pain severity, measured by Numeric pain rating scale (NPRS). All patients were followed for 1 week following enrollment.

**Results:**

The mean ages in the PNB group and control group were 58.6 and 57.4, respectively. One patient in the control group developed radiographic vasospasm. Three patients in both groups had radiographic hydrocephalus and IVH, requiring external ventricular drain (EVD) placement. The PNB group had an average reduction in mean raw pain score of 2.76 (4.68, 1.92 *p* = 0.024), and relative pain score by 0.26 (0.48, 0.22 *p* = 0.026), compared to the control group. The reduction occurred immediately after PNB administration.

**Conclusion:**

PNB can be a safe, feasible and effective treatment modality for HASH. Further investigations with a larger sample size are warranted.

## Introduction

Headache after aneurysmal subarachnoid hemorrhage (HASH) is common, severe, and refractory to conventional treatments (1, 2). HASH is often described initially as worst headache of life, and can linger in the background for weeks to months (2). The pathophysiology of headache in aneurysmal SAH (aSAH) remains poorly understood. Initial headache is thought to be from mechanical nociceptor stretching in vascular endothelium and raised intracranial pressure, (3) while sustained headache is thought to be a result of multifactorial activation of the trigeminal-vascular system (3–5). If untreated, HASH can potentially raise ICP due to dysregulated vasodilation by neural activation (6), which can worsen secondary brain injury (SBI). It can also hinder rehabilitation efforts by reducing participation, sleep, mood (7) and negatively impact quality of life (8).

HASH often persists through the time window of delayed cerebral ischemia (DCI) from aSAH, therefore it is imperative that treatment of HASH does not interfere with DCI detection. As it stands, analgesia modalities are limited in these patients. Current pharmacological treatment strategies including acetaminophen, gabapentin, magnesium, steroids, and non-steroidal anti-inflammatory agents (NSAIDs). Each modality is ineffective on its own, and many have significant drawbacks including clouding of neurological status, respiratory suppression and dose-dependent hepatotoxicity or nephrotoxicity. Despite high prevalence and potential deleterious effects of HASH, there is a lack of evidence-based treatment modalities for this disease with only 9% of providers indicating use of a standardized approach for HASH management, for which opioids remain the mainstay (9). Pain trajectories following aSAH are associated with continued opioid use at outpatient follow up (10), which contributes to the ongoing opioid epidemic. Pterygopalatine fossa blockade, recently reported by Smith et al. have recently emerged as a therapeutic option (11), but there continues to be an enormous need for an effective opioid-sparing treatment options in these patients, and more research is needed in this area.

Peripheral nerve blocks (PNBs) have been effective in treatment of acute and chronic headache disorders that involve the trigeminalovascular system, such as migraines, neuralgias, tension-type and chronic headaches (12–14). PNBs reduce headache intensity, duration and opioid usage (14, 15). Recently, a case report described success of a greater occipital nerve block for HASH in two patients (16). We report our pilot before-and-after observational controlled study, which compared patients receiving a bundle of bilateral supraorbital, greater and lesser occipital nerve blocks in addition to medical therapies, with patients receiving medical therapies alone for treatment of refractory HASH. Our hypothesis was that PNBs are safe, feasible, and will reduce HASH severity measured by the numeric pain rating scale (NPRS) immediately, and 1 week after administration.

## Methods

### Study design

We conducted a before-and-after observational study on a sample of 10 patients with refractory headache after aneurysmal subarachnoid hemorrhage admitted to the neurocritical care unit (NCCU). Headaches were classified as acute headache attributed to non-traumatic subarachnoid hemorrhage according to the 3rd edition of the International Classification of Headache Disorders (ICHD-3). The control group (*n* = 5) received standard-of-care medical treatments according to our HASH treatment protocol, and the intervention group (*n* = 5) received a standardized bundle of bilateral supraorbital, greater, and lesser occipital peripheral nerve blocks, in addition to standard-of-care treatments. The study was conducted between May 2019 and April 2020 at a large tertiary care teaching hospital. Data analysis was retrospective for the control group and prospective for the intervention group. The Institutional Review Board of West Virginia University Hospitals approved this study (IRB approval #1904522761 on 05/23/2019, “Peripheral Nerve Blocks for headache management in Subarachnoid Hemorrhage Population”), including a waiver of consent for the retrospective control group. Procedures were followed in accordance with institutional ethical standards and with the Helsinki Declaration of 1975. Informed consent was obtained from all participants in the prospective group. Inclusion criteria was as follows: ages 18 years or older, confirmed radiographic aSAH, Hunt and Hess grading of 1 or 2, ability to participate in informed consent procedure and presence of a refractory moderate or greater severity headache as defined by NPRS. Pain scores were documented according to the NPRS ranging from 0 to 10 by an intensive care unit nurse. Refractory headache was defined as a moderate or severe headache that persisted or worsened despite two modalities of treatment. Exclusion criteria included history of allergy to any of the medications in the protocol, coagulopathy not amenable to correction, recent suboccipital craniectomy, clinical or radiographic evidence of vasospasm at time of enrollment, history of cirrhosis or acute hepatic failure. Out of 30 patients that were screened, only 5 met inclusion criteria. The most common reason for not meeting inclusion criteria were a Hunt and Hess classification grading of greater than 2 and inability to participate in the informed consent procedure. During the enrollment period, all patients that met inclusion criteria consented for the PNB. All patients in the intervention group received a bundle of PNBs within 24 h of enrollment. All patients were treated for aSAH according to institutional protocols and international guidelines, including definitive aneurysm treatment within 24 h, administration of oral nimodipine and triweekly transcranial doppler ultrasonography (TCD) for vasospasm surveillance.

### Pain management

**Figure 1:**
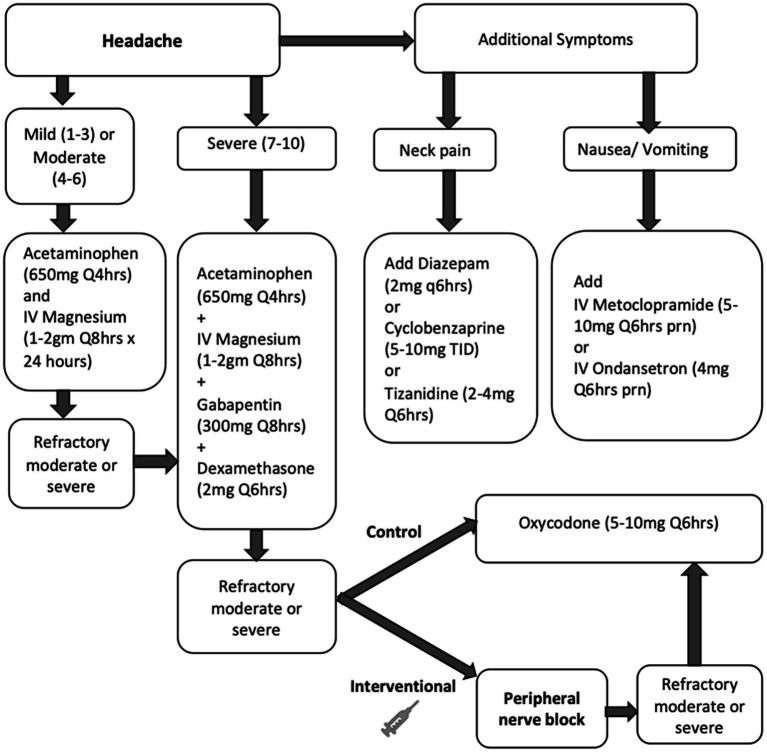
Study flow chart for treatment of HASH in the control (medications only) and intervention (peripheral nerve block) groups. Unless specified, medications were administered orally. Refractory headache was defined as a moderate or severe headache that persisted or worsened in intensity 1 h after treatment.

All patients in the study were treated with our standardized multimodal protocol for HASH management, as outlined in Figure 1. Initial treatment included acetaminophen doses up to a maximum of 4 grams daily and intravenous magnesium boluses 1–2 grams, not to exceed 4 grams total in 24 h, followed by scheduled gabapentin and dexamethasone for refractory headaches. Muscle relaxants and anti-emetics were used as adjunct therapy as needed. If HASH remained refractory to this treatment, treatment with opioids was initiated at a starting dose of 5 mg oxycodone every 6 h as needed in the control group and PNBs were administered in the intervention group. All PNBs were administered by two personnel in this study to limit variability, one neurointensivist and one trainee under close supervision of the same neurointensivist. If headaches were refractory to PNB, opioids were also allowed in the intervention group.

### Outcome and data collection

The primary outcome was pain severity, measured by the numeric pain rating scale (NPRS). The secondary outcome was adverse effects including bleeding, infection, hair loss or any reported adverse event due to the PNB or medications used in the trial. We performed a between-group comparison for the outcomes. In the control group, NPRS scores were documented immediately at time of protocol initiation, followed by every 4 h thereafter for 48 h. In the intervention group, NPRS scores were documented immediately before and after the procedure, followed by every 4 h for 48 h. Both groups had a one-time follow up NPRS documented 1 week after enrollment. Data were obtained from electronic medical record (EMR) in the retrospective group and recorded in a Health Insurance Portability and Accountability Act (HIPAA) compliant spreadsheet for the prospective group. Variables included age, Hunt and Hess score, need for mechanical ventilation, NPRS scores, presence or absence of external ventricular drain (EVD), aneurysm treatment and technique, hydrocephalus, intraventricular hemorrhage, or vasospasm during the clinical course, time from SAH to PNB placement, length of stay, complications and disposition. All medications administered were obtained from the medication administration record (MAR) in the EMR. If the patient had left the neurocritical care unit, a member of the research team contacted the patient in person or *via* telephone to obtain the one-week follow up score. There were no protocol changes or deviations during the study period.

### Peripheral nerve block procedure

**Figure 2:**
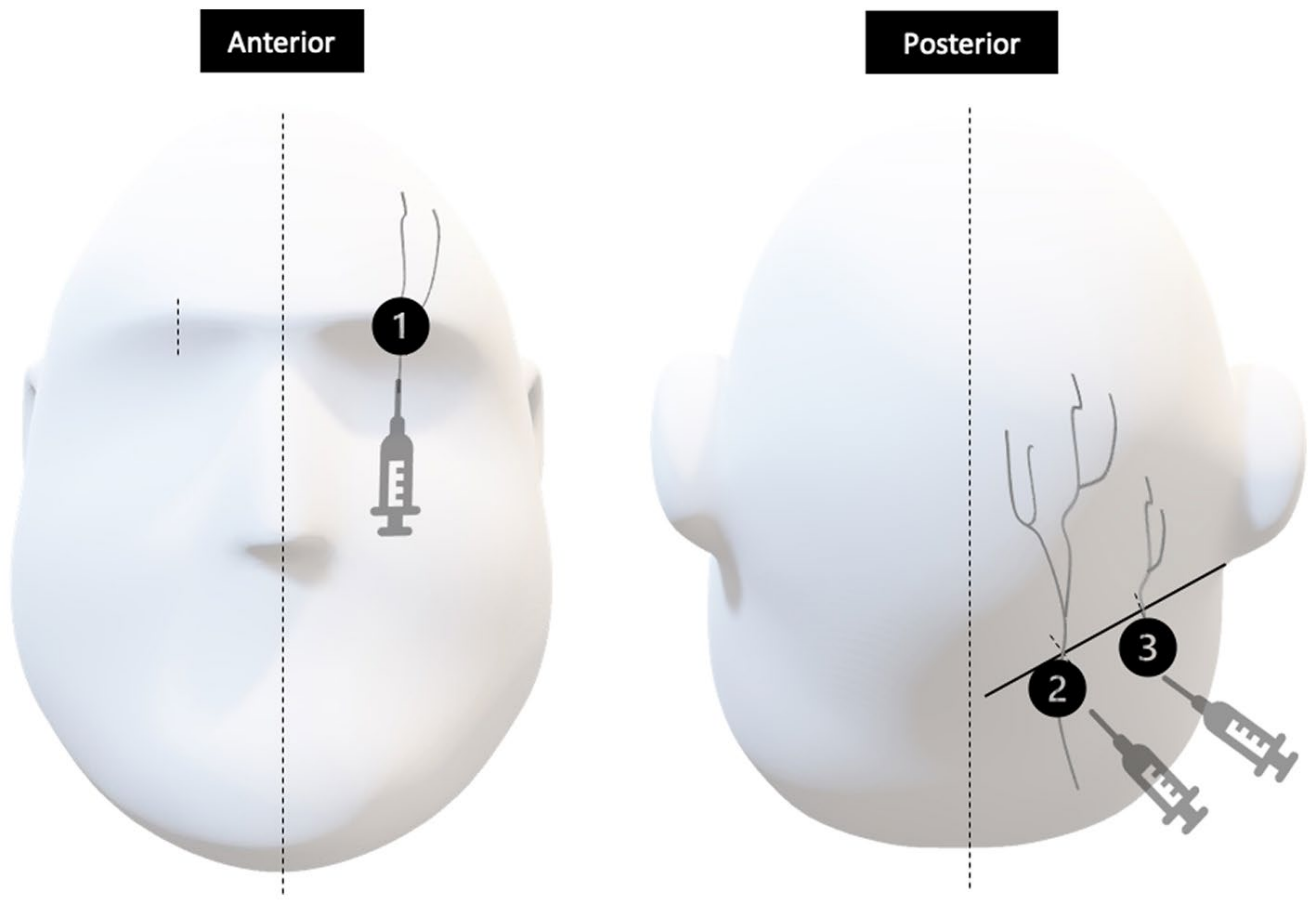
Injection sites represented by 1, 2, and 3 correspond to the supraorbital nerve, greater occipital nerve and lesser occipital nerves, respectively. In the anterior view, the supraorbital nerve is identified at about the mid-pupillary line. In the posterior view, greater and lesser occipital nerves exit at approximately one third and two thirds the distance between the external occipital protuberance and the mastoid process, respectively.

Once HASH was deemed at least moderate in severity and refractory to our standardized treatment algorithm as outlined in Figure 1, all patients in the prospective group participated in an informed consent procedure and received a PNB bundle at the bedside. Our PNB bundle consisted of bilateral greater occipital, lesser occipital and supraorbital nerve blocks, depicted in Figure 2. Each occipital nerve injection consisted of 3 ml of an equal mixture of 0.5% 5 mg/ml bupivacaine and 4 mg/ml dexamethasone. Each supraorbital nerve injection consisted of 2 ml of 0.5% 5 mg/ml bupivacaine.

### Statistical analysis

We used a repeated-measures ANOVA to model pain scores over time in both the intervention group that received the PNB and the control group. We used a Wilcoxon signed-rank test to compare the pre- and post-intervention pain scores. For testing significance, we assumed normality was violated due to the small sample size and used a bootstrap approach in which data are resampled to generate an empirical distribution of the test statistics. For this pilot study, no sample size was determined *a priori* so all consecutive patients fulfilling the inclusion criteria were included during the period. All statistical analysis was performed using R version 4.1.0 and RStudio version 1.3.959. The level of significance was set at 0.05 for all analyses.

## Results

Ten patients were included, 5 in the control group and 5 in the intervention (PNB) group. Their demographics are shown in Table 1. The mean ages in the control and PNB groups were 57.4 (median = 59, IQR = 12) and 58.6 (median = 60, IQR = 10), respectively. Mean Hunt and Hess scores in the control and PNB groups were 2.2 (median = 2, IQR = 0) and 2.4 (median = 2, IQR = 0), respectively and mean GCS scores in non-intubated patients were 14.25 in both groups (Control median = 14, IQR = 0.25 and intervention median = 14.5, IQR = 1.25). Three patients in both groups had radiographic hydrocephalus, 4 patients in the control group and 3 patients in the intervention group had IVH, requiring external ventricular drain (EVD) placement. None of the patients in either group underwent ventriculoperitoneal shunting. One patient in the control group developed radiographic vasospasm and 1 patient in each group was intubated during their hospital course in the NCCU. Five patients in the control group and 4 patients in the intervention group underwent aneurysm coiling, while 1 patient in the intervention group underwent aneurysm clipping. Three patients in the control group and 2 patients in the intervention group were discharged home, 2 patients in the control group and 3 patients in the intervention group were discharged to an acute rehabilitation facility. Mean time from aSAH to PNB placement was 7.2 days (median = 6, IQR = 2) in the intervention group. The mean ICU length of stay was 10.6 days in the control group (median = 10, IQR = 2) and 12.8 days in the intervention group (median = 14, IQR = 3). The mean hospital length of stay was 14.2 days in the control group (median = 14, IQR = 0) and 16.2 days in the intervention group (median = 16, IQR = 3). There was no difference in other aspects of management of these patients during the trial period.

**Table 1:** Demographic variables in control and intervention groups.

Variable		Control group, *n* = 5	Intervention group, *n* = 5
Age, years (Mean, median, IQR)		57.4, 59, 12	58.6, 60, 10
HH Score (Mean, median, IQR)		2.2, 2, 0	2.4, 2, 0
GCS on admission (Mean, median, IQR)	Intubated	10 T, 10 T, 10 T	10 T, 10 T, 10 T
	Not intubated	14.25, 14, 0.25	14.25, 14.5, 1.25
EVD Placement	No	2 (40%)	2 (40%)
	Yes	3 (60%)	3 (60%)
Hydrocephalus	No	2 (40%)	2 (40%)
	Yes	3 (60%)	3 (60%)
IVH	No	1 (20%)	2 (40%)
	Yes	4 (80%)	3 (60%)
Aneurysm treatment and technique	Coiling	5	4
	Clipping	0	1
Ventriculo-peritoneal shunting		0 (0%)	0 (0%)
Vasospasm	No	4 (80%)	5 (100%)
	Yes	1 (20%)	0 (0%)
Intubated	No	4 (80%)	4 (80%)
	Yes	1 (20%)	1 (20%)
Time from SAH to PNB placement, days (Mean, median, IQR)		N/A	7.2, 6, 2
Complications		0 (0%)	0 (0%)
ICU Length of Stay, days (Mean, median, IQR)		10.6, 10, 2	12.8, 14, 3
Hospital Length of Stay, days (Mean, median, IQR)		14.2, 14, 0	16.2, 16, 3
Disposition	Home	3 (60%)	2 (40%)
	Acute rehab	2 (40%)	3 (60%)
	Skilled nursing facility	0 (0%)	0 (0%)

HH, Hunt and Hess; GCS, Glasgow Coma Scale; IQR, Interquartile range; EVD, External ventricular drain; IVH, Intraventricular hemorrhage; SAH, subarachnoid hemorrhage; PNB, peripheral nerve block; ICU, Intensive care unit. Percentages out of group total have been provided for categorical variables.

**Figure 3:**
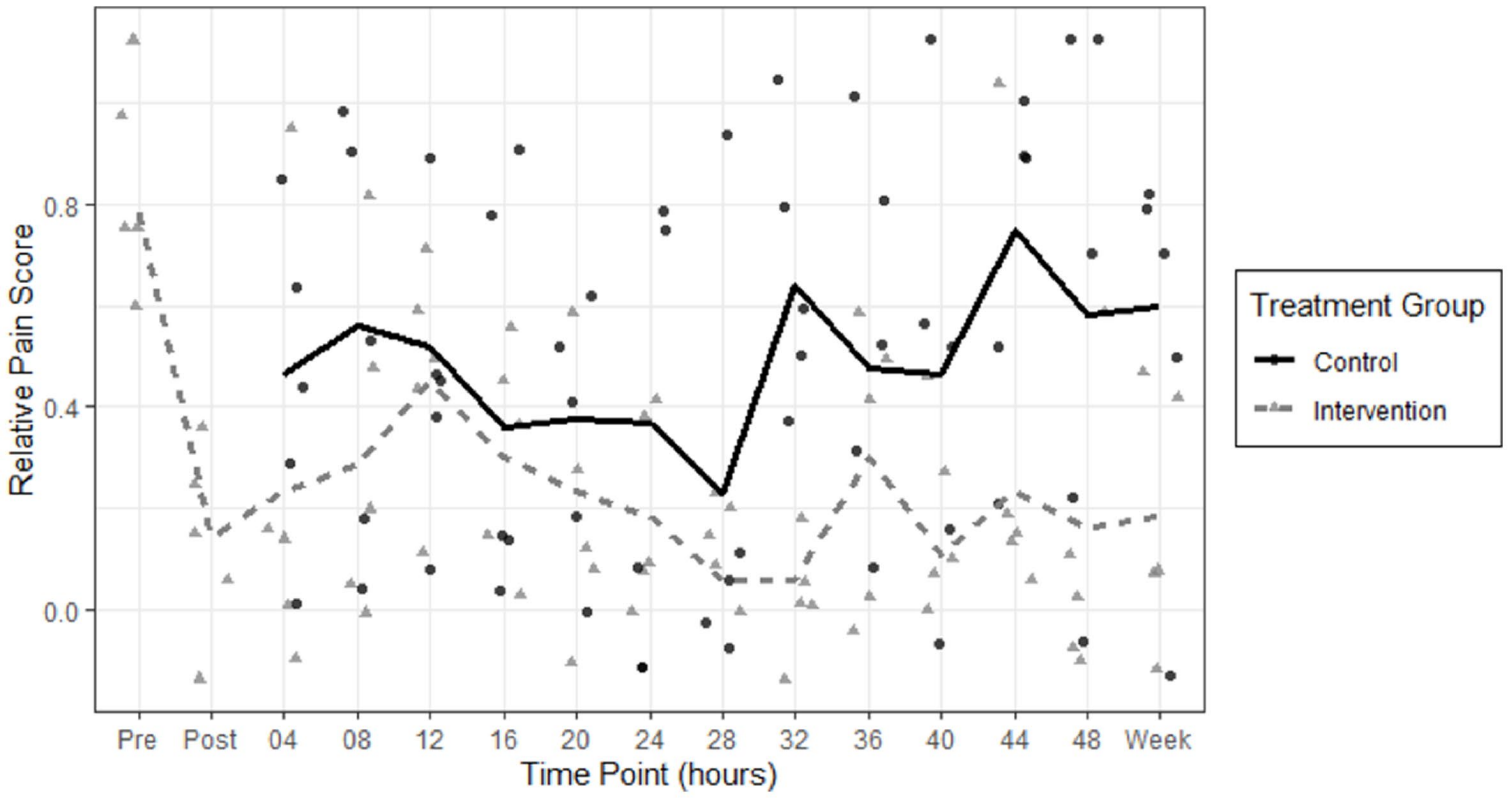
Scatterplot of relative NPRS pain scores in the control group (circles) and intervention peripheral nerve block (triangles) groups are shown over 48 h and on one-week follow up. A solid line shows the mean pain scores of the control group, and a dashed line shows the mean pain scores of the intervention group. The pain scores in the intervention group decrease immediately after the peripheral nerve blocks and remain lower over time, compared to the control group.

To account for variation in patient perception and tolerance of pain when using the raw NPRS, we calculated a scaled variable named relative NPRS by subtracting minimum recorded NPRS from raw NPRS and divided by maximum recorded NPRS for each patient. This transformed each patient’s score into a scaled value between 0 for minimum pain and 1 for maximal reported pain. We calculated the comparison of pain scores in both groups from the first time point after intervention, which was at 4 h. From 4 h post-intervention to 1 week, the PNB group had a mean raw pain score of 1.92, and the control group had a mean raw pain score of 4.68 (difference 2.76, *p* = 0.024). From 4 h post-intervention to 1 week, the PNB group had a mean relative pain score of 0.22, and the control group had a mean raw pain score of 0.48 (difference 0.26, *p* = 0.026). The biggest reduction in mean pain scores in the PNB group was between the pre- and post- periods after the PNB was administered, from 0.783 to 0.142 (difference 0.64, *p* < 0.003) and this reduction was sustained at 1 week upon follow-up. The results are demonstrated in a scatterplot of relative NPRS pain scores in both groups over time (Figure 3). A scatterplot of raw pain scores is also included in Supplementary Figure. A solid line shows the mean pain scores of the control group, and a dashed line shows the mean pain scores of the intervention group. The pain scores in the intervention group decrease immediately after the peripheral nerve blocks and remain lower over time, compared to the control group.

We did not find a further significant decrease in pain score at any of the time points compared to the initial decrease post-intervention (*p* = 0.9900). These results are shown in Figure 3.

There were no deviations from our standardized multimodal protocol for HASH management in the control group. Two patients in the intervention group received one-time doses of NSAIDs. One patient in the intervention group received opioids (67.5 morphine equivalent dose) for generalized pain including refractory HASH, prior to enrollment in the study. This patient had a chronic opioid dependence, with an active pre-hospitalization opioid prescription. Of note, this patient did not require opioids for the remainder of her hospitalization. Two patients in the control group received opioids for HASH. Given the small sample size and low incidence of opioid usage for HASH in our cohort, there was no meaningful difference among the two groups in opioid usage (Supplementary Table). There were no reported adverse effects in either group, including bleeding, infection, hair loss or transaminitis due to either PNB or medications used in this study.

## Discussion

In this small before-and-after case series of patients with refractory moderate or severe HASH, we found that a one-time bundle of supraorbital, greater occipital and lesser occipital peripheral nerve blocks performed at the bedside to be safe and feasible, as well as efficacious, compared to our standardized medical treatment approach. The relief was immediate and persisted up to a week on our intervention group of 5 patients, suggesting that PNB may a promising adjunct to oral or intravenous medications for patients with HASH, and a valuable treatment modality to study in a large prospective randomized controlled trial. To our knowledge, this is the first prospective study describing efficacy of PNB to treat HASH.

HASH is common, occurs in a majority of aSAH patients and is difficult to treat. In a cohort of aSAH patients, Morad et al. showed that 89% of patients reported a severe headache during their hospitalization (2). Improvements in emergency medical services, aneurysm treatment options and SBI prevention-focused intensive care unit (ICU) management have improved survival and functional outcomes in aSAH patients (17, 18). Despite these improvements, and a high prevalence of HASH, research in this area is lacking including, phenotypic HASH data, efficacious treatment options and treatment guidelines for providers. In the first cross-sectional worldwide study on HASH, Maciel et al. reported that HASH is recognized as a major clinical concern in 87% of providers, opioids are perceived as the most effective analgesic modality, and nearly half the providers prescribe opioids at discharge (9). This is problematic, given the drawbacks of opioid usage in the acute phase of injury following aSAH, as well as longer term, given the high potential for addiction and contribution to the ongoing opioid epidemic. Despite opioid usage, HASH remains unrelieved during hospitalization, with no single agent contributing to substantial HASH relief (19). Research on novel treatment options for HASH is greatly needed.

An effective treatment strategy for HASH likely needs to be multifaceted considering complex pathophysiology, individual pain perception and pain trajectories (10). Although exact mechanism of HASH has not been elucidated, current evidence suggests that blood in the subarachnoid space in the brain and neck can mechanically stretch and irritate trigeminovascular and greater occipital afferent neurons resulting in headache and meningismus (16). Trigeminal release of vasoactive peptides such as calcitonin gene-related peptide (CGRP) can result in vasodilation and activate inflammatory cascades, resulting in sustained activation of the trigeminal sensory system (5) and more prolonged HA (20). This can be compounded by ICP changes as well as cortical spreading depolarizations after SAH (21) that can disrupt ionic gradients, increase neuronal excitability, and further activate the trigeminal sensory system (5). Trigeminal and cervical sensory afferents converge in the spinal trigeminocervical complex (TCC), which gives rise to second-order trigeminothalamic tract neurons. Treatments targeting afferent input into the TCC, such as local perineural application of analgesia and steroids through PNB, may reduce afferent input into and excitability of second-order neurons. Such treatments may decrease central sensitization and alleviate both frontal and occipital HASH (16, 22, 23). Robust animal models have been used to understand HA pathophysiology including trigeminal sensory processing, and animal treatment models have led to substantial advancement in HA therapeutics (24).

Though numerous HASH treatment options exist, nearly all of them have disadvantages that limit their usage in the aSAH population, many having dose-dependent side effects. Acetaminophen and magnesium can be useful adjuncts but are often ineffective on their own (25). Risk of hepatic injury with doses greater than 3–4 grams a day limits dosing of acetaminophen in patients with inadequate HASH relief. Combination therapies with butalbital and caffeine are commonly associated with medication-overuse headaches if discontinued, therefore not ideal choices. Opioids can be sedating thus mask subtle neurological changes, falsely raise alarms for worsening neurological examinations resulting in unnecessary testing, produce hypercapnia (2) or hypotension that can be deleterious in dysregulated brain and compound secondary brain injury. In addition, opioids can result in nausea, vomiting, ileus, or urinary retention which again can raise ICP, contribute to medication-overuse headaches as well as long term dependence and addiction. Gabapentin and anti-epileptic drugs can be good adjuncts and work synergistically to reduce opioid dosing but can also reduce level of arousal in patients monitored closely for DCI (3, 26, 27). A majority of these adverse effects are dose-dependent. Triptans, serotonin 1B/1D receptor agonists and CGRP antagonists may be poor choices as they vasoconstrict cerebral vasculature (6), have case reports of association with reversible cerebral vasoconstriction syndrome and can theoretically worsen DCI in aSAH patients (28). The use of CGRP-modulating therapies in acute conditions such as traumatic brain injury and SAH continues to be explored (29, 30). Data on the effectiveness and safety of NSAIDs are limited in HASH, with over 50% of providers stating they rarely or never used NSAIDS in this setting (9), likely due to perceived risk of worsening bleeding. Systemic dexamethasone is often reserved for refractory cases due to systemic adverse effects of hyperglycemia, reduced sleep, and possible interference with wound healing. Effective HASH treatments are needed, and more non-pharmacological treatment approaches are needed to limit dose-dependent side effects of many of the current treatments.

PNBs are efficacious for treatment of a variety of headaches including occipital neuralgias, migraines, tension headaches and cervicogenic headaches (12, 14, 31), that share similar pathophysiological basis. Efficacy, toxicity and drug content of nerve blocks have been studied using mice models (32). In a case report composed of two patients with HASH, greater occipital PNB was found to provide effective analgesia and significantly reduced the need other medications (16). Smith et al. found bedside pterygopalatine fossa blockade to be efficacious for treatment of HASH and described their findings (11). Data from our observational study indicates that using PNB for HASH may be similarly feasible and effective. Our pilot study is the first to our knowledge that investigated PNBs to treat HASH and aims to add to the limited literature that exists in this important field.

Our study has numerous strengths, including prospective nature of the intervention arm and strict adherence to a standardized treatment protocol in both groups, so there was minimal confounding from dosages or timing of other treatments. Given variation in perception in pain among the general population, using an individualized pain score in addition to raw pain scores is also a strength. All patients were followed up at 1 week after enrollment, so we were able to show there was still benefit at that time point. Importantly, there is growing research that patients with HASH follow discrete pain trajectories (10), and pain trajectories can predict generation of chronic pain (9, 33). In our preliminary study, we found that PNB appeared to alter this trajectory immediately after administration, therefore it is plausible that it may alter and development of chronic HASH in some patients. The ability to perform PNBs at the bedside without specialized equipment allows this treatment modality to be used even in resource-scare environments. Many anesthesia and neurology residencies in North America provide procedural training in PNBs, therefore ease of locating a credentialed healthcare provider with relevant training and expertise may allow more widespread use of this modality to treat HASH. Scalp blocks including peripheral nerve blocks are routinely placed in the operating room by anesthesiologists and/or neurosurgeons for awake craniotomies and are considered low-risk (34).

Limitations of this before-and-after study include single-center observational study design combining retrospective (control group) and prospective data, as well as small sample size. We suspect that the small sample size may have played a role in being unable to detect a significant difference in opioid usage among the two groups. Despite strict adherence to a standardized protocol in both groups, uncaptured differences between the study periods or unblinded nature of the study may have contributed to differences in outcomes, including a placebo effect in the intervention group. It is also possible that patients that received a PNB may have been part of a discrete HASH pain trajectory cohort, that would have experienced improvement in pain scores at 7 days regardless of interventions. Headache phenotypes were not recorded in this study, which are important for future HASH studies. Only short-term 1 week follow-up information was available in our study group. We limited enrollment to patients that could communicate pain scores to us verbally, therefore our study population was limited to lower severity aSAH patients. Larger studies should be carried out on higher-grade aSAH patients that are able to endorse headaches to increase generalizability. Though we did not utilize repeat PNBs or alternate local anesthetics in patients with recurrent refractory HASH, this should be a consideration in future studies.

### Conclusion

Our small before-and-after observational study suggests that peripheral nerve blocks can be a safe, feasible and effective treatment option for headaches after aneurysmal subarachnoid hemorrhage. Further prospective investigations with a larger sample size are necessary.

## Key points

### Question

Do peripheral nerve blocks help to relieve refractory headaches after subarachnoid hemorrhage (HASH)?

### Findings

HASH is a global challenge among survivors, with a few effective treatment options. Peripheral nerve blocks appear feasible, reduce pain immediately after administration and provides sustained pain relief for up to a week compared to conventional medical treatment.

### Meanings

PNBs can be conducted safely at the bedside, appears feasible and efficacious for treatment of refractory HASH in our small before-and-after observational study.

## Data availability statement

The raw data supporting the conclusions of this article will be made available by the authors, without undue reservation.

## Ethics statement

The studies involving human participants were reviewed and approved by the Institutional Review Board of West Virginia University Hospitals approved this study (IRB approval #1904522761 on 05/23/2019, “Peripheral Nerve Blocks for headache management in Subarachnoid Hemorrhage Population”), including a waiver of consent for the retrospective control group. The patients/participants provided their written informed consent to participate in this study.

## Author contributions

SR: conceptualization, investigation, methodology, project administration, supervision, writing—original draft, and writing—review and editing. NS: data curation, project administration, writing—original draft, and writing—review and editing. AN: investigation, project administration, and writing—review and editing. JG: methodology, resources, and writing—review and editing. CJ: data curation, formal analysis, methodology, software, validation, visualization, and writing—review and editing. All authors contributed to the article and approved the submitted version.

## Conflict of interest

The authors declare that the research was conducted in the absence of any commercial or financial relationships that could be construed as a potential conflict of interest.

## Publisher’s note

All claims expressed in this article are solely those of the authors and do not necessarily represent those of their affiliated organizations, or those of the publisher, the editors and the reviewers. Any product that may be evaluated in this article, or claim that may be made by its manufacturer, is not guaranteed or endorsed by the publisher.

## Supplementary material

The Supplementary material for this article can be found online at: https://www.frontiersin.org/articles/10.3389/fneur.2023.1122384/full#supplementary-material

## References

[1] Swope R Glover K Gokun Y Fraser JF Cook AM. Evaluation of headache severity after aneurysmal subarachnoid hemorrhage. Interdiscipl Neurosurg. (2014) 1:119–2. doi: 10.1016/j.inat.2014.07.003

[2] Morad AH Tamargo RJ Gottschalk A. The longitudinal course of pain and analgesic therapy following aneurysmal subarachnoid hemorrhage: a cohort study. Pain. (2016) 56:1617–25. doi: 10.1111/head.12908 27704534

[3] Dhakal LP Harriott AM Capobianco DJ Freeman WD. Headache and its approach in Today's NeuroIntensive care unit. Neurocrit Care. (2016) 25:320–4. doi: 10.1007/s12028-016-0260-z, PMID: 27000642

[4] van Lieshout JH Dibué-Adjei M Cornelius JF Slotty PJ Schneider T Restin T. An introduction to the pathophysiology of aneurysmal subarachnoid hemorrhage. Neurosurg Rev. (2018) 41:917–08. doi: 10.1007/s10143-017-0827-y, PMID: 28215029

[5] Ashina H Porreca F Anderson T Amin FM Ashina M Schytz HW. Post-traumatic headache: epidemiology and pathophysiological insights. Nat Rev Neurol. (2019) 15:607–17. doi: 10.1038/s41582-019-0243-8, PMID: 31527806

[6] Kofke WA Rajagopalan S Ayubcha D Balu R Cruz-Navarro J Manatpon P. Defining a taxonomy of intracranial hypertension: is ICP more than just a number? J Neurosurg Anesthesiol. (2020) 32:120–1. doi: 10.1097/ana.0000000000000609, PMID: 31135572 PMC6872925

[7] Tassorelli C Tramontano M Berlangieri M Schweiger V D’Ippolito M Palmerini V. Assessing and treating primary headaches and cranio-facial pain in patients undergoing rehabilitation for neurological diseases. J Headache Pain. (2017) 18:99. doi: 10.1186/s10194-017-0809-z, PMID: 28963668 PMC5622014

[8] Huckhagel T Klinger R Schmidt NO Regelsberger J Westphal M Czorlich P. The burden of headache following aneurysmal subarachnoid hemorrhage: a prospective single-center cross-sectional analysis. Acta Neurochir. (2020) 162:893–3. doi: 10.1007/s00701-020-04235-7, PMID: 32016589 PMC7066282

[9] Maciel CB Barlow B Lucke-Wold B Gobinathan A Abu-Mowis Z Peethala MM. Acute headache Management for Patients with subarachnoid hemorrhage: an international survey of health care providers. Neurocrit Care. (2022):1–12. doi: 10.1007/s12028-022-01571-7 [Epub ahead of print]. 35915347

[10] Jaffa MN Jha RM Elmer J Kardon A Podell JE Zusman BE. Pain trajectories following subarachnoid hemorrhage are associated with continued opioid use at outpatient follow-up. Neurocrit Care. (2021) 35:806–4. doi: 10.1007/s12028-021-01282-5 34109554 PMC8189709

[11] Smith CR Fox WC Robinson CP Garvan C Babi MA Pizzi MA. Pterygopalatine fossa blockade as novel, narcotic-sparing treatment for headache in patients with spontaneous subarachnoid hemorrhage. Neurocrit Care. (2021) 35:241–8. doi: 10.1007/s12028-020-01157-1, PMID: 33403584

[12] Perloff MD Chung JS. Urgent care peripheral nerve blocks for refractory trigeminal neuralgia. Am J Emerg Med. (2018) 36:2058–60. doi: 10.1016/j.ajem.2018.08.019, PMID: 30119988

[13] Allen SM Mookadam F Cha SS Freeman JA Starling AJ Mookadam M. Greater occipital nerve block for acute treatment of migraine headache: a large retrospective cohort study. J Am Board Fam Med. (2018) 31:211–8. doi: 10.3122/jabfm.2018.02.170188, PMID: 29535237

[14] Hascalovici JR Robbins MS. Peripheral nerve blocks for the treatment of headache in older adults: a retrospective study. Headache. (2017) 57:80–6. doi: 10.1111/head.12992, PMID: 27901275

[15] Cardwell TW Zabala V Mineo J Ochner CN. The effects of perioperative peripheral nerve blocks on Peri- and postoperative opioid use and pain management. Am Surg. (2022) 88:2842–50. doi: 10.1177/00031348211023395, PMID: 34162251

[16] Doğan R Pınar HU Karaca Ö Karakoç F. Ultrasound-guided bilateral greater occipital nerve block on headache seen after endovascular treatment of ruptured or unruptured intracranial aneurysms: a case report. Agri. (2018) 32:223–7. doi: 10.5505/agri.2018.59023 33398867

[17] Lovelock CE Rinkel GJ Rothwell PM. Time trends in outcome of subarachnoid hemorrhage: population-based study and systematic review. Neurology. (2010) 74:1494–01. doi: 10.1212/WNL.0b013e3181dd42b3, PMID: 20375310 PMC2875923

[18] Roquer J Cuadrado-Godia E Guimaraens L Conesa G Rodríguez-Campello A Capellades J. Short- and long-term outcome of patients with aneurysmal subarachnoid hemorrhage. Neurology. (2020) 95:e1819–29. doi: 10.1212/wnl.0000000000010618, PMID: 32796129 PMC7682825

[19] Viswanathan V Lucke-Wold B Jones C Aiello G Li Y Ayala A. Change in opioid and analgesic use for headaches after aneurysmal subarachnoid hemorrhage over time. Neurochirurgie. (2021) 67:427–2. doi: 10.1016/j.neuchi.2021.03.006, PMID: 33771620

[20] Karatas H Erdener SE Gursoy-Ozdemir Y Lule S Eren-Koçak E Sen ZD. Spreading depression triggers headache by activating neuronal Panx1 channels. Science. (2013) 339:1092–5. doi: 10.1126/science.1231897, PMID: 23449592

[21] Dreier JP. The role of spreading depression, spreading depolarization and spreading ischemia in neurological disease. Nat Med. (2011) 17:439–7. doi: 10.1038/nm.2333 21475241

[22] Jürgens TP Müller P Seedorf H Regelsberger J May A. Occipital nerve block is effective in craniofacial neuralgias but not in idiopathic persistent facial pain. J Headache Pain. (2012) 13:199–3. doi: 10.1007/s10194-012-0417-x, PMID: 22383125 PMC3311831

[23] Castien R De Hertogh W. A neuroscience perspective of physical treatment of headache and neck pain. Front Neurol Perspective. (2019) 10:e00276. doi: 10.3389/fneur.2019.00276 PMC6443880 30972008

[24] Harriott AM Strother LC Vila-Pueyo M Holland PR. Animal models of migraine and experimental techniques used to examine trigeminal sensory processing. J Headache Pain. (2019) 20:91. (In eng). doi: 10.1186/s10194-019-1043-7, PMID: 31464579 PMC6734323

[25] Dorhout Mees SM Bertens D van der Worp HB Rinkel GJ van den Bergh WM. Magnesium and headache after aneurysmal subarachnoid haemorrhage. J Neurol Neurosurg Psychiatry. (2010) 81:490–3. doi: 10.1136/jnnp.2009.181404, PMID: 19828484

[26] Dhakal LP Hodge DO Nagal J Mayes M Richie A Ng LK. Safety and tolerability of gabapentin for aneurysmal subarachnoid hemorrhage (sah) headache and meningismus. Neurocrit Care. (2015) 22:414–1. doi: 10.1007/s12028-014-0086-5, PMID: 25403765

[27] Dhakal LP Turnbull MT Jackson DA Edwards E Hodge DO Thottempudi N. Safety, tolerability, and efficacy of pain reduction by gabapentin for acute headache and Meningismus after aneurysmal subarachnoid hemorrhage: a pilot study. Front Neurol. (2020) 11:744. doi: 10.3389/fneur.2020.00744, PMID: 32849209 PMC7399216

[28] Rozen TD Bhatt AA. Reversible cerebral vasoconstriction syndrome developing after an erenumab injection for migraine prevention. Cephalalgia. (2022) 42:250–6. doi: 10.1177/03331024211037277, PMID: 34405713

[29] Mehkri Y Hanna C Sriram S Lucke-Wold B Johnson RD Busl K. Calcitonin gene-related peptide and neurologic injury: an emerging target for headache management. Clin Neurol Neurosurg. (2022) 220:107355. doi: 10.1016/j.clineuro.2022.107355, PMID: 35785661

[30] Kokkoris S Andrews P Webb DJ. Role of calcitonin gene-related peptide in cerebral vasospasm, and as a therapeutic approach to subarachnoid hemorrhage. Front Endocrinol (Lausanne). (2012) 3:135. doi: 10.3389/fendo.2012.00135, PMID: 23162536 PMC3498620

[31] Patel D Yadav K Taljaard M Shorr R Perry JJ. Effectiveness of peripheral nerve blocks for the treatment of primary headache disorders: a systematic review and meta-analysis. Ann Emerg Med. (2022) 79:251–1. doi: 10.1016/j.annemergmed.2021.08.007, PMID: 34756448

[32] Shikanov A Domb AJ Weiniger CF. Long acting local anesthetic-polymer formulation to prolong the effect of analgesia. J Control Release. (2007) 117:97–3. doi: 10.1016/j.jconrel.2006.10.014, PMID: 17137669

[33] Ha H Gonzalez A. Migraine headache prophylaxis. Am Fam Physician. (2019) 99:17–24. 30600979

[34] Chaki T Sugino S Janicki PK Ishioka Y Hatakeyama Y Hayase T. Efficacy and safety of a Lidocaine and Ropivacaine mixture for scalp nerve block and local infiltration anesthesia in patients undergoing awake craniotomy. J Neurosurg Anesthesiol. (2016) 28:1–5. doi: 10.1097/ana.0000000000000149, PMID: 25493926

