# Supplementary material for: Safety and efficacy of peripheral nerve blocks to treat refractory headaches after aneurysmal subarachnoid hemorrhage – A pilot observational study
Source: Front Neurol. 2023 Apr 20;14:1122384. doi: 10.3389/fneur.2023.1122384 (PMC10158792; doi:10.3389/fneur.2023.1122384)
Supplement: Supplementary file 1 [file Table_1.docx]

**Supplementary Table:**

| **Outcomes** | **Control group** | **Interventional group** | **p-value** |
| --- | --- | --- | --- |
| Average relative pain score | 0.48 | 0.22 | 0.026 |
| Adverse effects | 0 | 0 | N/A |
| Number of subjects that used opiates | 2 | 1 | N/A |

Outcome data recorded for the control group and the interventional peripheral nerve block groups. The average relative pain score was lower in the interventional group. There were no adverse effects reported. There was 1 subject in each group that used opiates.
