# Supplementary material for: Safety and efficacy of peripheral nerve blocks to treat refractory headaches after aneurysmal subarachnoid hemorrhage – A pilot observational study
Source: Front Neurol. 2023 Apr 20;14:1122384. doi: 10.3389/fneur.2023.1122384 (PMC10158792; doi:10.3389/fneur.2023.1122384)
Supplement: Supplementary file 2 [file Image_1.pdf]

Supplementary Figure

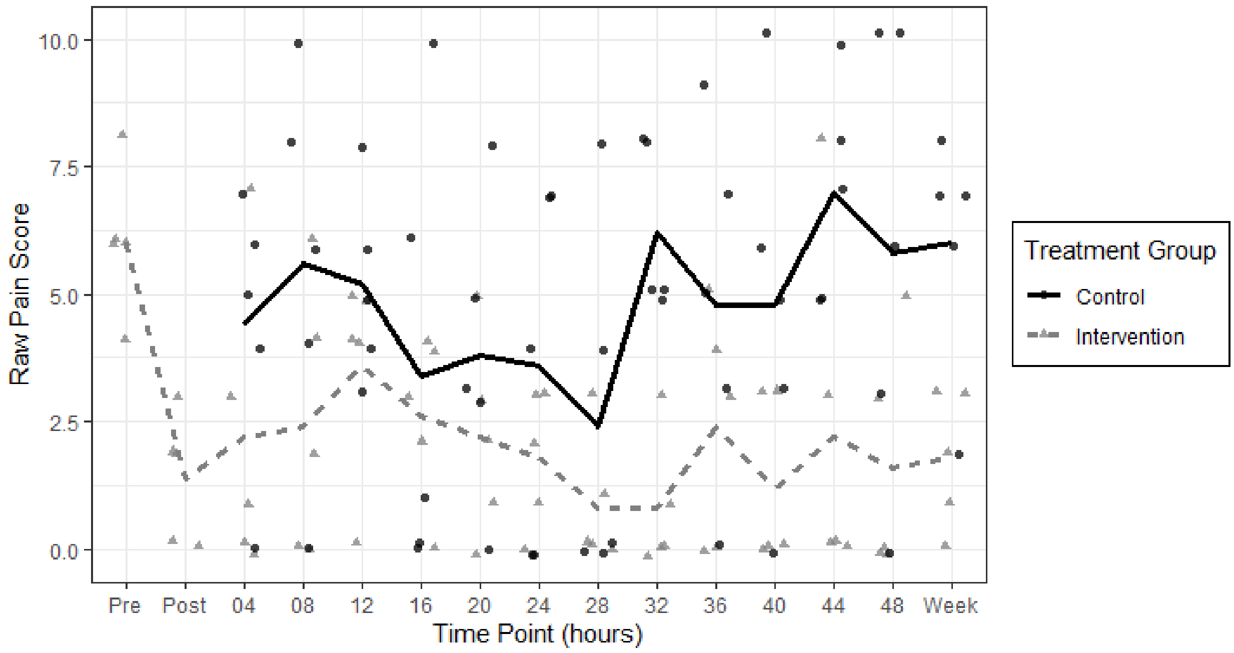

Scatterplot of raw NPRS pain scores in the control medication-only (circles) and interventional peripheral nerve block (triangles) groups are shown over a period of 48 hours and on one week follow up. A solid line shows the mean pain scores of the control group, and a dashed line shows the mean pain scores of the interventional group.
